# X-linked lymphoproliferative disease type 1 (XLP1) due to a “de novo” missense SH2D1A Hemizygous Mutation Leading to Predominantly Antibody Deficiency

**DOI:** 10.21203/rs.3.rs-6649535/v1

**Published:** 2025-06-25

**Authors:** Andrés F. Zea-Vera, Sebastián Giraldo-Ocampo, Mónica Fernandes-Pineda, Anilza Bonelo-Perdomo, Elizabeth M. Kang

**Affiliations:** LCIM, NIAID, NIH Bethesda; Universidad del Valle; Universidad del Valle; Universidad del Valle; LCIM, NIAID, NIH Bethesda

**Keywords:** XLP1, CVID, lymphoproliferative disorder, hypogammaglobulinemia

## Abstract

**Purpose:**

The SAP-SLAM receptor system plays a critical role in immune regulation, with SAP deficiency leading to apoptosis resistance in T cells and disrupting immune homeostasis, as seen in XLP-1. This disorder, characterized by uncontrolled lymphoproliferation, often presents with a variable clinical spectrum.

**Methods:**

We report a case of XLP-1 with dysgammaglobulinemia in a patient initially diagnosed with pediatric-onset CVID. Through quantitative and functional analysis of the **SH2D1A** c.164G > A (p.Arg55Gln) variant, we confirmed its pathogenicity.

**Results:**

Our findings demonstrate that this variant significantly impairs B cell differentiation and proliferation independently of T cell interactions

**Conclusion:**

These results support the pathogenic nature of **SH2D1A** R55Q and advocate for its reclassification as a pathogenic mutation.

## Introduction

X-linked lymphoproliferative disease type 1 (XLP1) is a rare Inborn Error of Immunity (IEI) caused by mutations in the SH2D1A/SAP gene that causes an exaggerated immune response to Epstein-Barr virus (EBV) infections [1]. XLP1 is characterized by three main phenotypes: fulminant infectious mononucleosis (FIM)/ hemophagocytic lymphohistiocytosis (HLH); B-cell lymphoma and/or lymphoproliferation; and a milder phenotype which presents with dysgammaglobulinemia that can progress to hypogammaglobulinemia and is associated with recurrent infections [2, 3]. In addition to these various phenotypes, all patients have reduced or absent iNKT cells, reduced memory B cells, reduced NK and CTL cytotoxic activity and up to 3% of cases may present with aplastic anemia, necrotizing vasculitis, lymphoid pulmonary granulomatosis, or eosinophilia [2-4]. The most common presenting feature of XLP1 is FIM/ HLH, especially during primary EBV infection, with a frequency of 39.6% and a mortality rate of up to 80%. The second most common, developing during the illness is dysgammaglobulinemia with a frequency of 22% and mortality up to 5% [5].

The *SH2D1A* gene that encode the SLAM-associated protein (SAP) [6], is expressed mainly on T, NK, and NKT cells [3]. SLAM family receptors are expressed only on hematopoietic cells and consist of six members [7]. When SLAM family receptors are activated, SAP protein is recruited to their cytoplasmic domain preventing the coupling of phosphatases to the receptor. Furthermore, SAP recruits the Src-related protein tyrosine kinase Fyn and the downstream signal transduction participates in the activation and adhesion of NK and T cells to their targets [7]. However, in SAP-deficient patients SLAM receptors become potent inhibitory receptors once activated due to the recruitment and coupling of phosphatases [3, 7].

The diagnosis of XLP1 is based on the sequencing of the *SH2D1A* gene, although blood lymphocyte SAP expression can be measured by flow cytometry or western blot analysis as patients have an absent or reduced amount of this protein [8]. SAP analysis, quantification of iNKT cells and T cell restimulation-induced cell death analysis can also be used as supporting diagnostic assays [5, 9]. However relevant biological processes such as B cell differentiation or activation are not well described. Not all clinical manifestations are EBV infection-dependent [5], making diagnosis more difficult without molecular testing. As such patients are mistakenly diagnosed under the common variable immunodeficiency (CVID) umbrella [10]. Moreover, some variants found in *SH2D1A* are reported without functional or laboratory analysis and thus classified as variants of uncertain significance (VUS). We present the quantitative and functional evaluation of a “*de novo”* hemizygous variant in the *SH2D1A* gene (c.164G > A p.Arg55Gln) in a young male, with significant clinical sequelae.

## Materials and methods

### Human Subjects

The patient and his relatives provided written informed consent in accordance with the Declaration of Helsinki under institutional review board – approved protocols of Universidad del Valle, Cali, Colombia. Blood from healthy donors and patients was obtained under approved protocols, which also allowed for the collection and use of patients’ family history and pedigrees for publication.

### Blood samples and isolation of Peripheral Blood Mononuclear Cells (PBMC)

20 mL of anti-coagulated peripheral blood was collected by venipuncture into EDTA vacutainer tubes. 500uL was used for hemogram analysis; the remaining blood was used for PBMC isolation, genetic analysis, SAP assessment, and iNKT quantification.

PBMCs were isolated by density gradient centrifugation using Ficoll-Paque (Sigma-Aldrich). Briefly, total blood was diluted in 10 mL of RPMI 1640 and centrifuged at 2000 rpm with 3 mL of Ficoll-Paque. The upper layer containing the plasma was removed and the PBMC fraction was transferred into a new 15 mL tube and washed twice with 10mL RPMI 1640. Cell viability and counting were performed using trypan blue staining. PBMC were cryopreserved in liquid nitrogen in freezing medium (40% FBS/10% DMSO) until further analysis.

### Genetic evaluation and in silico analysis

Sequence analysis and deletion/duplication testing of peripheral total blood cells DNA from the patient was analyzed at Invitae Corporation (https://www.invitae.com), San Francisco, through the 207-primary immunodeficiency-associated genes. The variant found in the SH2D1A gene was confirmed by direct sequencing using the Sanger method and tested in his brother and mother as well.

**Figure 1:**
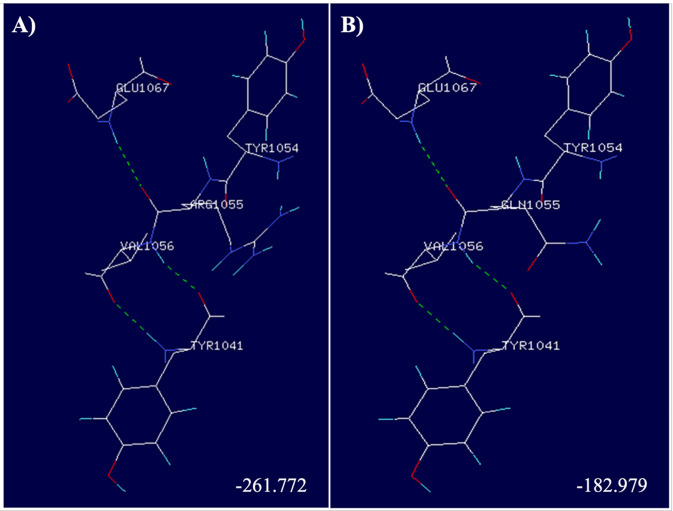
Crystal structure of a segment of SAP protein. WT arginine in position 55 (ARG1055) forms three hydrogen bonds, represented as green dashed lines (A). There are no changes of this hydrogen bonds when the Glutamine (GLN1055) replaces the WT arginine (B).

The functional impact of the variant was predicted in PROVEAN + SIFT, mutation assessor. and SuSPect predictor software. Changes in Gibbs free energy and hydrogen bonds were evaluated in the protein crystal structure using Swiss PDB Viewer software (crystal reference in protein data bank: 1D1Z). (Fig. 1)

### Flow cytometry analyses

Immunophenotyping of B cell subsets was performed in PBMCs by flow cytometry using the pregerminal center (pre-GC) antibody panel proposed by the EuroFlow Consortium [11]: CD19- PECy7 (clone J3-119) from Beckman Coulter; CD5-PE (clone UCHT-2), SmIgD-PerCPCy5.5 (clone IA6-2) and SmIgM-BV510 (clone MHM-88) from Biolegend; CD21-APC (clone B-ly4), CD27-BV421 (clone M-T271) and CD38-HB7 from BD Biosciences. Based on the staining pattern of the above-mentioned antibodies and the side (SSC) and forward scatter (FSC) parameters, B cells (CD19^+^ SSC^low^ FSC^low^ lymphocytes) were classified into immature/transitional B lymphocytes (CD5^+^CD27^−^CD38^++^smIgM^++^smIgD^+^); naïve CD5^+^ (CD5^+^CD27^−^CD38^+ d^smIgM^+^smIgD^++^) and CD5^−^ (CD5^−^CD27^−^CD38^+ d^smIgM^+^ smIgD^++^) B lymphocytes and their CD21^+^ and CD21^−^ subsets; switched memory B cells (CD5^−^CD27^+/−^CD38^−^smIgM^−^smIgD^−^); unswitched memory B cells (CD5^−^CD27^+^CD38^−^smIgM^+^ smIgD^++^) and plasmablasts (CD5^−^CD27^++^CD38^+++^). A minimum of 2x10^6^ PBMC were used and stained for 30 minutes at room temperature with the corresponding antibodies at optimal concentration. Data acquisition was performed with the FACSCanto^™^ II flow cytometer (Beckman Coulter) and analyzed using FlowJo^™^ v10.07 Software (BD Life Sciences).

iNKT quantification was performed using the antibodies CD3-PerCP and TCRVα24-FITC (clone 6B11, Biolegen). SAP expression was assessed by using the antibodies, CD3, CD8, CD56, and intracellular staining with SAP-PE (clone 1A9, Merck Millipore) after fixation and permeabilization of the cells. Both analyses were performed in total blood by technical services at the Primary Immunodeficiencies research group, Universidad de Antioquia, Colombia. All the analyses included one age and gender-matched healthy control.

### In vitro B cell activation, plasmablast generation and CFSE lymphoproliferation

*In vitro* analysis was carried out by adapting protocols previously described[12, 13]. Briefly, 4x10^6^ PBMC were thawed, washed, and suspended in PBS, and incubated with 0.125 μM carboxyfluorescein succinimidyl ester (CFSE, Invitrogen, Cat. #C34570) for 5 min at room temperature. Then, CFSE labelled-PBMC were centrifuged at 1500 rpm for 5 min, resuspended in complete media (RPMI 1640, 10% bovine fetal serum), and cultured for 6 days at 37°C with 5% CO2 at a concentration of 2x10^5^ cells/200μL/well in flat-bottomed plates in the presence of 10 μg/mL anti-CD40 (Invitrogen, Catalog #16-0409-81) and 50 ng/mL IL-21 (Gibco, Cat. #PHC0211); 1.25 μM CpG/ODN 2006 (Miltenyi Biotec, Cat. #130-100-106) or complete medium without stimuli. The same culture conditions were performed with non CFSE labelled-PBMC for plasmablast frequency assessment. B cells proliferation (i.e., dilution of CFSE) and the frequency of CD19^+^CD27^++^CD38^+++^ plasmablasts was evaluated by flow cytometry. Three healthy controls were included and the experiments were performed in duplicate.

## Results

Index patient is a 28-years-old Colombian male born from healthy unrelated parents with healthy siblings (half-brother and half-sister). At the age of 8, the patient presented with a right lower lobe persistent pneumonia that required partial lobectomy. The pathology report did not report any additional findings. Between 9 and 14-years of age, the patient presented with 3 bacterial pneumonias that required hospitalization for antibiotic treatment without oxygen requirement, etiological causes undetermined although blood, sputum and even BAL cultures were done.

At age 15, in the context of an admission for pulmonary tuberculosis, he was evaluated by clinical immunology, and found to have hypogammaglobulinemia (IgM: 9, IgG: 430 and IgA:16 mg/dL), absent anti-HB S Ag, rubella, or mumps IgG response (despite prior vaccination) and preserved B cells 3,2% of leukocytes. A diagnosis of Common Variable Immunodeficiency (CVID) was given, and intravenous immunoglobulin (800mg/Kg every month) was initiated. Lung CT-scan revealed disseminated cystic bronchiectasis (BGI score = 12) with severely affected lung function. Cystic fibrosis and primary ciliary dyskinesia were ruled out.

**Figure 2:**
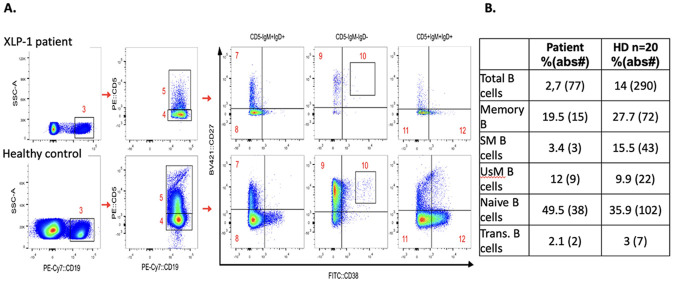
A. Gating strategy for B cells subsets evaluation comparing XLP1 patient PBMCs with a representative healthy control B. Percentage % and absolute number (abs#) o B cell subsets in peripheral blood of the XLP1 patient compare with the mean values in n=20 healthy donors

At the age of 21, during a pulmonary tuberculosis relapse, patient required admission to the ICU for ventilatory support. At that moment peripheral blood showed a normal absolute T cell count (2568cells/uL) with CD4 + T cell lymphopenia (556cells/uL) and an inverted CD4/CD8 ratio (0.3), absolute B cells were low (70.9cells/uL 2,3%) and NK cells were normal (183cells/uL). Lung function decay, spirometry showed: FVC 28%, FEV1 16% and FEV1/FVC 49%, thus a diagnosis of COPD requiring O2 was stablished. In addition to B cell lymphopenia, B cell subsets showed a relative increase in naïve B cells and unswitched memory B cell frequencies and low switched memory B cells compatible with a B cell pattern #3 characterized by B-cell activation and proliferation defects (Fig. 2).

**Figure 3:**
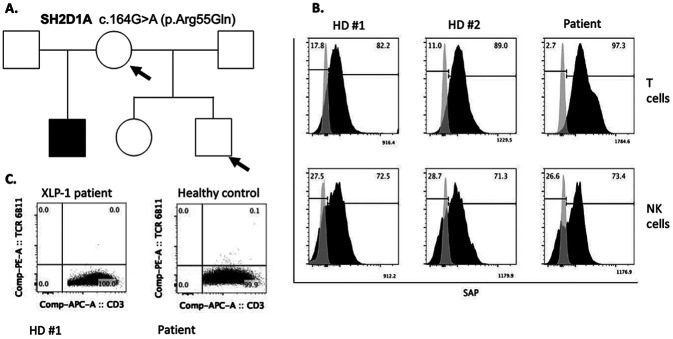
A. Plasmablast differentiation after 6 days in culture with CpG/ODN 2006 upper or CD40L+IL21 lower dot plots B. CFSE dilution gated in CD19+ cells (B cell proliferation) after 6 days in culture: unstimulated, CpG/ODN 2006 or CD40L+IL21 C. Plasmablasts frequencies and D. divided cells CD19+ in the patient (red dots) as compare with 3 Healthy controls (blue dots) after 6 days in culture.

At age of 24 year, a genetic Panel (207 genes) (courtesy of the Jeffrey Modell Foundation) was performed. A novel missense *SH2D1A* hemizygous mutation c.164G > A (p.Arg55Gln) was found. Sanger sequencing confirmed the presence of the variant in SH2D1A gene in the patient but not in his mother or half-brother stablishing the “*de novo”* status of the variant (Fig. 3A). *In silico* analysis of the variant in PROVEAN, SIFT, mutational assessor and SusPect predicted a deleterious (score=−3.68), damaging (score = 0), medium (FI score = 2.18) and disease-associated (score = 59/100) functional impact, respectively. The predicted Combined Annotation Dependent Depletion (CADD score) was 31. Due to the association with serious complications related with EBV and the futility of serological evaluation since the patient receives replacement immunoglobulins that contain anti-EBV antibodies, viral load for EBV was performed in plasma and PBMCs and were found to be negative.

Although the “*in silico”* prediction was deleterious, according with ACMG/AMP guidelines and the provider (INVITAE) the variant was reported as of uncertain significant (VUS). We proceeded to evaluate the expression of SAP in T cells and NK cells finding a similar frequency and mean fluorescence intensity as compare with healthy controls (Fig. 3B) indicating the variant does not affect protein stability. However, iNKT cells were absent (Fig. 3C) and NK cytotoxicity assay using K562 cells as target cells was significantly reduced (data not shown). These data together suggest the pathogenicity of the variant.

**Figure 4:**
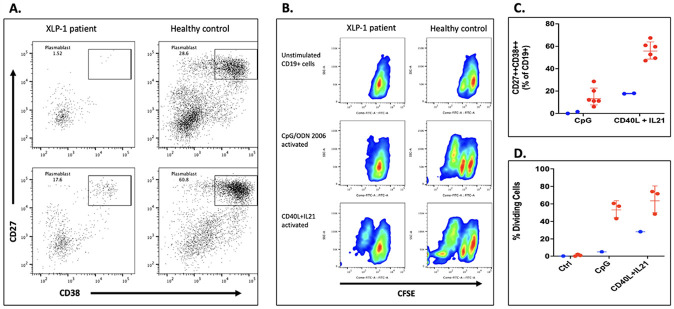
A. Plasmablast differentiation after 6 days in culture with CpG/ODN 2006 upper or CD40L+IL21 lower dot plots B. CFSE dilution gated in CD19+ cells (B cell proliferation) after 6 days in culture: unstimulated, CpG/ODN 2006 or CD40L+IL21 C. Plasmablasts frequencies and D. divided cells CD19+ in the patient (red dots) as compare with 3 Healthy controls (blue dots) after 6 days in culture.

Our findings support the pathogenic effect of this variant but do not explain the hypogammaglobulinemia and B cell lymphopenia. We evaluated B cell lymphoproliferation and differentiation to plasmablasts in response to T-dependent (CD40L + IL21) or T-independent (CpG/ODN 2006) stimuli after 3 or 6 days in culture. Patient’s PBMCs exhibited a significant reduction in differentiation to plasmablasts (Fig. 4A) and a severely reduced lymphoproliferation indicating profound impairment in the B cell compartment maturation. Interestingly, the patient was vaccinated at the beginning of 2021 against COVD-19 (Pfizer) and at the end of 2023 (Moderna) but his serum IgM and IgG level against SARS-CoV2 remained negative until the writing of this paper.

Currently the patient has worsening of respiratory symptoms, an increase in supplementary oxygen requirements, and still has recurrent bacterial pneumonia receiving intermittent azithromycin prophylaxis. Unfortunately, immunoglobulin supplementation has been irregular due to insurance issues combined with a human immunoglobulin (IVIG or SCIg) shortage in Colombia.

## Discussion

XLP-1 is a disorder characterized by impaired immune homeostasis, primarily due to apoptosis resistance in SAP-deficient T cells [5]. The SAP-SLAM receptor system plays a critical role in immune responses, including CD4 + T cell-mediated B cell help, CD8 + T cell and NK cell-mediated cytotoxicity, particularly against B cells, and regulation of CD8 + T cell apoptosis [14]. In XLP-1, the absence of functional SAP results in defective apoptosis of T cells, promoting their survival and subsequent lymphoproliferation, ultimately disrupting immune homeostasis [5, 14].

We report a case of XLP1 with dysgammaglobulinemia in a patient presenting with a pediatric-onset CVID-like phenotype. A quantitative and functional evaluation of the c.164G > A (p.Arg55Gln) variant in SH2D1A confirmed its pathogenicity. While this mutation has been previously described in two patients in separate reports [15, 16], this is the first study to perform both quantitative and functional assessments. Our findings indicate that despite normal SAP protein expression, the R55Q variant is associated with absent iNKT cells, B cell lymphopenia, reduced memory B cells, impaired NK and CTL cytotoxic activity, and significantly decreased B cell proliferation. Furthermore, this SH2D1A mutation prevents the in vitro generation of plasmablasts, contributing to dysgammaglobulinemia.

The patient was initially diagnosed as having pediatric-onset CVID, guiding the initial treatment approach [1]. However, comprehensive molecular testing and functional validation allowed us to reclassify the patient as having XLP-1 with dysgammaglobulinemia case, demonstrating that normal SAP expression does not necessarily correlate with normal functional activity. Each year, new immunodeficiency-related genes are identified, and genetic analysis enables more precise immunologic treatments [1]. In Latin America, access to diagnostic and genetic tools remains limited, however as shown here, their use can significantly alter prognosis and management [17]. This underscores the urgent need for comprehensive programs integrating NGS and functional validation in developing countries.

Several SLAM family receptors are highly expressed in B cells, which may contribute to the distinct phenotypes observed in XLP-1 [5]. These include defective germinal center formation, leading to dysgammaglobulinemia—often preceding EBV infection, as seen in this patient—impaired B cell elimination by cytotoxic mechanisms, increasing the risk of B-cell lymphomas, and an inability to control EBV infection due to the virus’s tropism for B cells.[14]

EBV-induced fulminant infectious mononucleosis (FIM) and hemophagocytic lymphohistiocytosis (HLH) remain the most common and life-threatening manifestations of XLP-1. The excessive accumulation of CD8 + effector T cells drives multi-organ damage in EBV-induced FIM/HLH, despite their defective cytotoxic response against EBV-infected B cells [14]. In this patient, the absence of detectable EBV in plasma and PBMCs suggests that management should prioritize regular monitoring for EBV reactivation, HLH, or lymphoma through periodic EBV DNA testing and clinical evaluations. In XLP-1, rituximab is mainly used to control EBV-related complications by depleting B cells; however, its routine use is not advised in the absence of active EBV infection due to the risk of unnecessary immunosuppression [18].

SAP is also essential for T cell restimulation-induced cell death (RICD), a self-regulatory mechanism crucial for maintaining T cell homeostasis [5]. Pharmacological inhibition of DGKα has been shown to restore RICD and prevent excessive virus-induced expansion of CD8 + T cells in laboratory mice, offering a potential therapeutic target for EBV-induced FIM in XLP-1 by restoring apoptosis sensitivity [19]. While DGKα inhibition presents a promising strategy, further studies are needed to evaluate its efficacy in cases of functional SAP alterations.

Additional analyses are needed to assess potential SAP protein dysfunction when suspected [7]. Clinical research plays a pivotal role in advancing laboratories dedicated to primary immunodeficiency diseases (PIDDs), which focus on diagnosing and understanding inherited immune disorders [20]. This type of investigation extends beyond inborn errors of immunity; importantly, targeting the SAP/NTB-A signaling pathway may also have therapeutic potential in cancer immunotherapy. In this context, NTB-A blockade has been shown to enhance CD8 + T cell responses in mouse tumor models, possibly by reducing RICD sensitivity [21].

Early transplantation is the preferred and only curative option for XLP-1. However, due to an initially mild phenotype, delayed diagnosis, and limited access to genetic testing, an increasing number of transplants are being performed later in young adults with primary immunodeficiencies. Early evaluation for transplantation is crucial to prevent severe complications and improve long-term outcomes, especially considering that approximately 80% of patients undergoing alloHSCT achieve complete cellular and humoral reconstitution [5].

The 2015 ACMG Evidence Framework for Variant Classification was developed to facilitate consistent communication regarding variant pathogenicity by integrating genetic evidence along a spectrum from benign to pathogenic [22]. In the present case, several in silico predictive tools were utilized to evaluate the SH2D1A R55Q variant. PROVEAN yielded a deleterious score of −3.68, SIFT classified the variant as damaging (score = 0), Mutational Assessor indicated a medium functional impact (FI score = 2.18), and SusPect assigned a disease-associated score of 59/100. Furthermore, the Combined Annotation Dependent Depletion (CADD) score was 31, placing the variant among those with high predicted pathogenic potential. These computational predictions were corroborated by functional assays, which revealed that the R55Q variant markedly disrupts B cell differentiation and proliferation, independent of T cell engagement. Taken together, these data strongly support the pathogenic nature of the SH2D1A R55Q variant and justify its reclassification as a pathogenic mutation.

## References

[1] Tangye SG, Al-Herz W, Bousfiha A, Human Inborn Errors of Immunity: 2022 Update on the Classification from the International Union of Immunological Societies Expert Committee. J Clin Immunol. 2022;42:1473–507. 35748970 10.1007/s10875-022-01289-3 PMC9244088

[2] Schwartzberg PL, Mueller KL, Qi H, Cannons JL. SLAM receptors and SAP influence lymphocyte interactions, development and function. Nat Rev Immunol. 2009;9:39–46. 19079134 10.1038/nri2456

[3] Cannons JL, Tangye SG, Schwartzberg PL. SLAM Family Receptors and SAP Adaptors in Immunity. Annu Rev Immunol. 2011;29:665–705. 21219180 10.1146/annurev-immunol-030409-101302

[4] Tangye SG. XLP: Clinical Features and Molecular Etiology due to Mutations in SH2D1A Encoding SAP. J Clin Immunol. 2014;34:772–9. 25085526 10.1007/s10875-014-0083-7

[5] Booth C, Gilmour KC, Veys P, X-linked lymphoproliferative disease due to SAP/SH2D1A deficiency: a multicenter study on the manifestations, management and outcome of the disease. Blood. 2011;117:53–62. 20926771 10.1182/blood-2010-06-284935 PMC3374620

[6] Panchal N, Booth C, Cannons JL, Schwartzberg PL. X-Linked Lymphoproliferative Disease Type 1: A Clinical and Molecular Perspective. Front Immunol. 2018. 10.3389/fimmu.2018.00666. PMC5893764 29670631

[7] Wu N, Veillette A. SLAM family receptors in normal immunity and immune pathologies. Curr Opin Immunol. 2016;38:45–51. 26682762 10.1016/j.coi.2015.11.003

[8] Filipovich AH, Zhang K, Snow AL, Marsh RA. X-linked lymphoproliferative syndromes: brothers or distant cousins? Blood. 2010;116:3398–408. 20660790 10.1182/blood-2010-03-275909 PMC2981470

[9] Chiang SCC, Bleesing JJ, Marsh RA. Current Flow Cytometric Assays for the Screening and Diagnosis of Primary HLH. Front Immunol. 2019. 10.3389/fimmu.2019.01740. PMC6664088 31396234

[10] Morra M, Silander O, Calpe S, Choi M, Oettgen H, Myers L, Etzioni A. (2001) Alterations of the X-linked lymphoproliferative disease gene SH2D1A in common variable immunodeficiency syndrome. 98:1321–5. 10.1182/blood.v98.5.1321 11520777

[11] Van Dongen JJM, Van Der Burg M, Kalina T. (2019) EuroFlow-Based Flowcytometric Diagnostic Screening and Classification of Primary Immunodeficiencies of the Lymphoid System. 10:1–21. 10.3389/fimmu.2019.01271 PMC6585843 31263462

[12] Recher M, Berglund LJ, Avery DT, IL-21 is the primary common ✉ chain-binding cytokine required for human B-cell differentiation in vivo. Blood. 2011;118:6824–35. 22039266 10.1182/blood-2011-06-362533 PMC3338166

[13] De Kerk DJ, Jansen MH, Jolles S, Warnatz K, Seneviratne SL, Berge IJM, Van Leeuwen EMM. (2016) Phenotypic and Functional Comparison of Class Switch Recombination Deficiencies with a Subgroup of Common Variable Immunodeficiencies. J Clin Immunol 656–66. 27484504 10.1007/s10875-016-0321-2 PMC5018261

[14] Pende D, Meazza R, Marcenaro S, Aricò M, Bottino C. 2B4 dysfunction in XLP1 NK cells: More than inability to control EBV infection. Clin Immunol. 2019;204:31–6. 30391652 10.1016/j.clim.2018.10.022

[15] Pachlopnik Schmid J, Canioni D, Moshous D, Clinical similarities and differences of patients with X-linked lymphoproliferative syndrome type 1 (XLP-1/SAP deficiency) versus type 2 (XLP-2/XIAP deficiency). Blood. 2011;117:1522–9. 21119115 10.1182/blood-2010-07-298372

[16] Miller PG, Niroula A, Ceremsak JJ, Identification of germline variants in adults with hemophagocytic lymphohistiocytosis. Blood Adv. 2020;4:925–9. 32150605 10.1182/bloodadvances.2019001272 PMC7065469

[17] Giugliani R. Inborn errors of metabolism in Latin America: challenges and opportunities. J Inherit Metab Dis. 2010;33:315–20. 20454860 10.1007/s10545-010-9112-8

[18] Korah-Sedgwick MM, Wall LA. (2018) EBV Infection in XLP1 Manifested Solely by Behavioral Aggression and Effective Treatment Using Rituximab. Case Reports Immunol 2018:3705376. 29977631 10.1155/2018/3705376 PMC6011099

[19] Ruffo E, Malacarne V, Larsen SE, Inhibition of diacylglycerol kinase α restores restimulation-induced cell death and reduces immunopathology in XLP-1. Sci Transl Med. 2016;8:321ra7. 10.1126/scitranslmed.aad1565 PMC4918505 26764158

[20] Rispoli F, Valencic E, Girardelli M, Immunity and Genetics at the Revolving Doors of Diagnostics in Primary Immunodeficiencies. Diagnostics. 2021;11:532. 33809703 10.3390/diagnostics11030532 PMC8002250

[21] Hajaj E, Eisenberg G, Klein S, SLAMF6 deficiency augments tumor killing and skews toward an effector phenotype revealing it as a novel T cell checkpoint. Elife. 2020. 10.7554/eLife.52539. PMC7075692 32122464

[22] Richards S, Aziz N, Bale S, Standards and guidelines for the interpretation of sequence variants: a joint consensus recommendation of the American College of Medical Genetics and Genomics and the Association for Molecular Pathology. Genet Med. 2015;17:405–24. 25741868 10.1038/gim.2015.30 PMC4544753

